# Comparison of clinical and pathological characteristics between early gastric cardiac cancer and early gastric non-cardiac cancer

**DOI:** 10.3389/fonc.2025.1513011

**Published:** 2025-07-22

**Authors:** Bin Yu, Hong-Lei Wu, Shuai Li, Dao-Yu Tao, Zhen-Xiang Zuo, Xing Qi, Hui-Min Zhang

**Affiliations:** ^1^ Department of Gastroenterology, The Second Hospital, Cheeloo College of Medicine, Shandong University, Jinan, Shandong, China; ^2^ Department of Pathology, The Second Hospital, Cheeloo College of Medicine, Shandong University, Jinan, Shandong, China

**Keywords:** early gastric cardiac cancer, early gastric non-cardiac cancer, clinical and pathological characteristics, invasion depth, incomplete resection

## Abstract

**Background:**

Previous studies primarily focused on advanced-stage gastric cancer have identified significant differences between gastric cardiac cancer (GCC) and gastric non-cardiac cancer (GNCC) in clinical, pathological, and molecular phenotypes. This study focuses on early-stage GCC and GNCC cases treated with Endoscopic Submucosal Dissection (ESD) to investigate differences in clinical presentation and case characteristics, thereby providing scientific evidence for early diagnosis and treatment strategies for gastric cancer.

**Methods:**

This study retrospectively analyzed 304 patients, comprising 126 with early GCC and 178 with early GNCC. To further investigate the differences between the early GCC and early GNCC, 1:1 propensity score matching was applied, enabling a more rigorous comparison of the endoscopic and clinicopathological features between the two cohorts. Patients were categorized based on tumor involvement in the deep submucosa (≥500 μm, SM2) and positive ESD margins. Multivariate analysis was conducted to identify independent risk factors for SM2 or deeper infiltration and incomplete resection.

**Results:**

Compared to the early GNCC group (n = 178), the early GCC group (n = 126) had a higher proportion of older patients and males. After propensity score matching, early GCC cases (n = 112) exhibited a greater prevalence of moderately differentiated tubular adenocarcinoma, a lower prevalence of papillary carcinoma, a higher likelihood of SM2 or deeper infiltration, and an increased probability of positive margins after ESD (P < 0.05 for all comparisons). Gastric cardia location (odds ratio (OR) = 3.395, P = 0.031), larger tumor size (OR = 1.375, P = 0.006), and mixed-type adenocarcinoma (OR = 6.975, P < 0.001) were identified as independent risk factors for SM2 or deeper infiltration in early gastric cancer. In early GCC cases, moderately differentiated tubular adenocarcinoma (OR = 14.959, P = 0.035) and SM2 or deeper invasion (OR = 16.65, P < 0.001) were identified as independent risk factors for positive margins following ESD.

**Conclusion:**

GCC and GNCC show notable differences in clinical and pathological characteristics, with GCC demonstrating higher invasiveness. Therefore, endoscopists should strengthen identification and intervention efforts for early-stage cancers in the cardia region to improve patient prognosis.

## 1 Introduction

China is a high-incidence region for Gastric cancer (GC), with 2022 statistics indicating that gastric cardiac cancer (GCC) and gastric non-cardiac cancer (GNCC) accounted for approximately 70% and 50% of global cases, respectively. Early-stage GC often presents asymptomatically, with most patients exhibiting no clinical signs, leading to diagnosis at more advanced stages. Research has demonstrated that the 5-year survival rate for early-stage GC can exceed 85%, while it drops below 30% for stage IV disease (1–3). Additionally, the prognosis for GCC is typically poorer (4–6). Thus, early detection and treatment of GC are critical (7). Endoscopic Submucosal Dissection (ESD) represents the current standard treatment for early GC, offering benefits such as fewer postoperative complications and faster recovery (8–10).

In 2000, the World Health Organization classified GCC as adenocarcinoma of the esophagogastric junction. According to the Siewert classification, GCC corresponds to Siewert type II adenocarcinoma, located 1 cm above and 2 cm below the gastroesophageal junction. However, the precise location of GCC remains somewhat ambiguous, generally referring to the narrow area of the proximal stomach just below the gastroesophageal junction. In 2010, the World Health Organization defined early-stage GC as a tumor confined to the mucosa or submucosa, regardless of lymph node metastasis. GCC and GNCC differ significantly in terms of site of occurrence, risk factors, and clinicopathological characteristics. Gene chip research has revealed notable differences in gene expression and signaling pathways between the two, supporting GCC as a distinct tumor category (11, 12). Over recent decades, with effective control of Helicobacter pylori (Hp), the incidence of GNCC has declined, while GCC incidence has gradually increased, with a trend of primary tumors shifting toward the proximal stomach, particularly in East Asian populations (4, 12).

This study systematically examined early GC patients who underwent ESD at a tertiary medical center in China. It analyzed the clinical, endoscopic, and pathological differences between early GCC and GNCC, with the aim of identifying independent risk factors for deep tumor invasion and positive margins following ESD.

## 2 Materials and methods

### 2.1 Patients

Patients with early GC who underwent ESD at the Second Hospital of Shandong University between January 2017 and June 2024, and whose diagnoses were confirmed by pathological examination, were included in this study. The study was conducted in accordance with the principles of the Declaration of Helsinki. All participants underwent gastroscopy using the GIF-HQ290, GIF-H260, and Olympus models. We typically use narrow-band imaging (NBI) with magnifying endoscopy to observe the structure and microvascular patterns on the surface of the lesion, confirm lesion boundaries, assess histological types, and estimate the invasion depth. After a detailed evaluation of the lesion, we mark approximately 2 mm outside the demarcation line between the tumor tissue and normal tissue under high magnification in NBI using strong coagulation (ERBE electrosurgical workstation, Effect 3, Power 50W). The pathology report is verified and reviewed by at least two pathologists. The inclusion criteria were: (1) early GC located in the gastric cardia or distal stomach (represented by the angular notch and pyloric antrum); (2) patients primarily treated with ESD; and (3) patients with complete medical records. The exclusion criteria were: (1) the presence of other systemic malignant tumors; and (2) severe comorbidities, such as cardiovascular diseases, hematological disorders, neuropsychiatric conditions, or liver and kidney dysfunction.

### 2.2 Data collection and grouping

Data were collected on the following variables: gender, age, smoking and alcohol history, tumor diameter (categorized based on whether the longest diameter was ≤3 cm), endoscopic macroscopic type [elevated type (I, IIa, IIa+IIc), flat type (IIb), depressed type (IIc, IIc+IIa, III)], histological type (Tub1: well-differentiated tubular adenocarcinoma; Tub2: moderately differentiated tubular adenocarcinoma; papillary adenocarcinoma (pap): ≥30% papillary structures; mixed-type adenocarcinoma: containing more than two histological types; signet ring cell carcinoma (SRCC): ≥50% signet ring cells), invasion depth (M (mucosa), SM1 (from the muscularis mucosa to the submucosa < 500 μm), SM2 or deeper), background mucosal status (none/mild to moderate intestinal metaplasia, severe intestinal metaplasia), ESD margin status (negative or positive), lymphovascular invasion, and other relevant factors. Patients were grouped by tumor location [GCC and GNCC (represented by the angular notch and pyloric antrum)], invasion depth (M/SM1, SM2 or deeper), and ESD margin status (negative or positive).

### 2.3 Statistical analysis

Data processing was conducted using IBM SPSS Statistics 27.0. Age, gender, smoking history, and alcohol consumption history were treated as confounding variables and matched in a 1:1 ratio using the nearest neighbor matching method. A caliper value of 0.02 was applied, resulting in a total of 224 matched cases. The standardized differences for all matched variables were below 10%. Continuous variables were described as mean (standard deviation, SD) or median (interquartile range, IQR), and differences were analyzed using t-tests or Wilcoxon rank-sum tests. Categorical variables were presented as counts and percentages, with comparisons made using the χ² test. Logistic regression analysis was employed to assess independent risk factors for infiltration depth and positive margins after ESD. Variables with P < 0.1 in univariate analysis were included in the multivariate analysis. Statistical significance was defined as P < 0.05.

## 3 Results

### 3.1 Demographic characteristics

**Figure 1:**
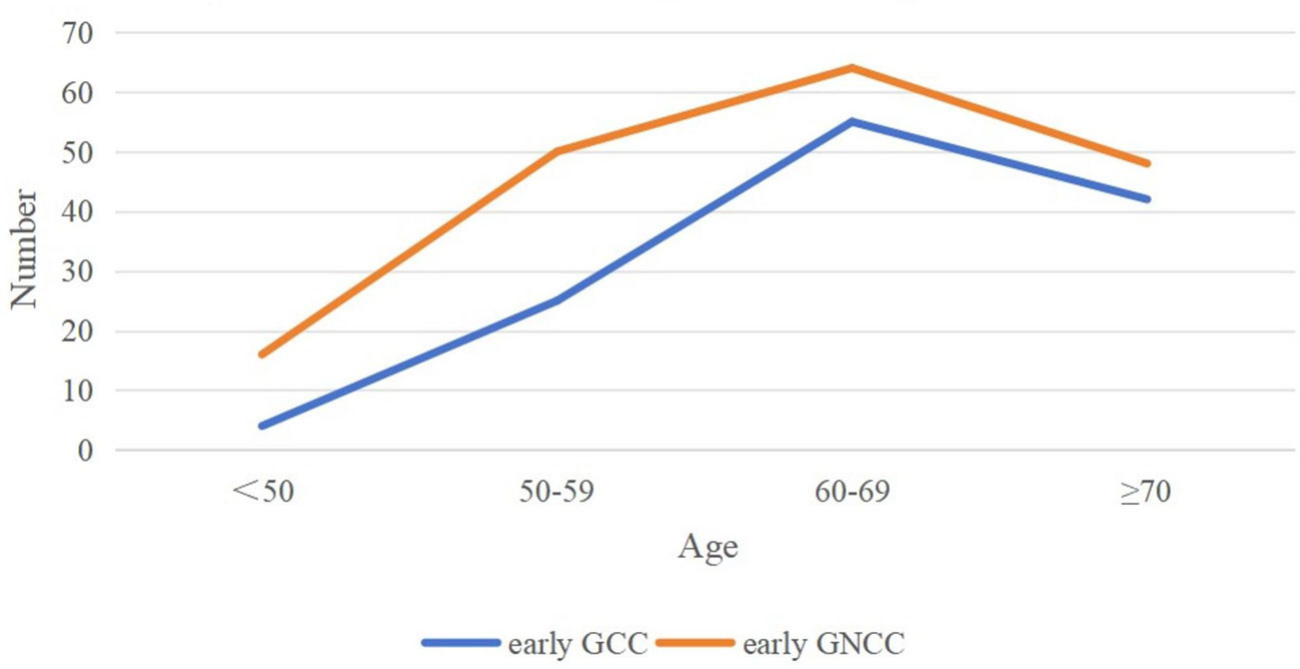
Age distribution trend of early GCC and early GNCC incidence.

Based on the inclusion and exclusion criteria, 304 patients with early GC were enrolled in the study. Among them, 126 patients were assigned to the early GCC group, and 178 patients were assigned to the early GNCC group. Patients in the early GCC group were significantly older on average than those in the early GNCC group (65.29 years vs. 62.90 years), and the proportion of males/female was higher (6.41 vs. 2.49), with both differences yielding P values of less than 0.05. There were no statistically significant differences between the two groups in terms of smoking or alcohol history (**Table 1**). Both groups exhibited peak incidence between the ages of 60 and 69 (**Figure 1**).

**Table 1:** Baseline characteristics of patients.

Baseline Characteristics	Early GCC (n=126)	Early GNCC (n=178)	χ^2^/*t*	*P* value
Age (Years), n (%)	65.29 ± 8.546	62.90 ± 9.353	*t*=2.255	0.024
Age group, n (%)			χ^2^ = 4.554	0.033
≤65	58 (56.0)	104 (58.4)		
>65	68 (54.0)	74 (41.6)		
Gender, n (%)			χ^2^ = 9.764	0.002
Male	109 (86.5)	127 (71.3)		
Female	17 (13.5)	51 (26.9)		
Male/Female Ratio	6.41	2.49		
Smoking status, n (%)			χ^2^ = 0.983	0.343
Never	69 (54.8)	106 (60.2)		
Former/ Current	57 (45.2)	70 (39.8)		
Alcohol use, n (%)			χ^2^ = 2.863	0.067
Never	68 (54.0)	114 (64.4)		
Former/Current	58 (46.0)	63 (35.6)		

### 3.2 Comparison of endoscopic features of early gastric cancer

**Figure 2:**
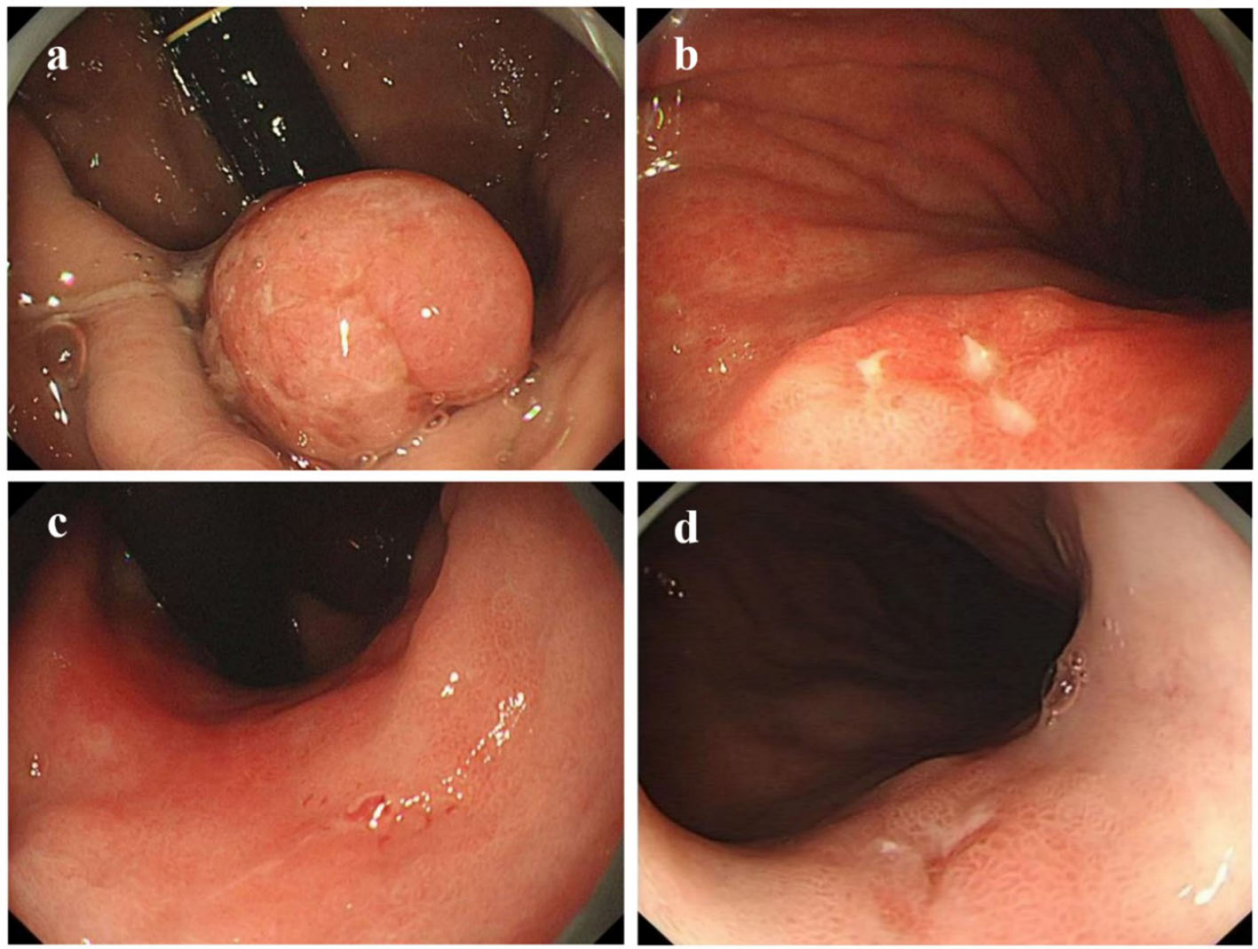
Gastric cancer Paris classification as seen under conventional white-light endoscopy (C-WLI). **(a)** Elevated Lesion (Paris Classification Type I): The lesion presents as a smooth, round, red elevation that protrudes significantly above the surrounding mucosa with distinct boundaries. **(b)** Slightly Elevated Lesion (Paris Classification Type IIa+IIc): The elevation is mild, with a smooth surface and a broader base. **(c)** Flat Lesion (Paris Classification Type IIb): The lesion is nearly level with the surrounding mucosa, showing minimal elevation. It blends into the surrounding tissue, making detection more challenging compared to elevated lesions. **(d)** Slightly Depressed Lesion (Paris Classification Type IIc): This lesion displays a clear depression or an area lower than the surrounding mucosa. The surface is slightly irregular, and the edges of the depression are well defined.

Using age, gender, smoking history, and alcohol history as confounding factors, a 1:1 matching procedure was conducted, resulting in a total of 224 cases. A descriptive analysis of the endoscopic characteristics of early GCC and early GNCC was conducted, followed by a differential analysis, as presented in **Table 2**. The gross morphology of early GC under traditional white-light endoscopy can be classified into three groups according to the Paris classification: elevated type (0-I, IIa, and IIa+IIc), flat type (0-IIb), and depressed type (0-IIc, III, and IIc+IIa) (see **Figure 2** for characteristics). In both patient groups, the depressed type was the most common, followed by the elevated type, while the flat type was the least common. The distribution was not statistically significant. For details, refer to **Table 2**.

**Table 2:** Comparison of endoscopic characteristics between the two groups.

Clinical endoscopy parameters	Early GCC (n=112)	Early GNCC (n=112)	χ^2^	*P* value
Macroscopic Classification, n (%)			χ^2^ = 2.986	0.225
elevated type	35 (31.3)	42 (37.5)		
flat type	22 (19.6)	13 (11.6)		
depressed type	55 (49.1)	57 (50.9)		
Endoscopic Color, n (%)			χ^2^ = 3.768	0.052
Reddish tone	110 (98.2)	104 (92.9)		
whitish tone	2 (54.0)	8 (7.1)		

### 3.3 Comparison of pathological features of early gastric cancer

**Figure 3:**
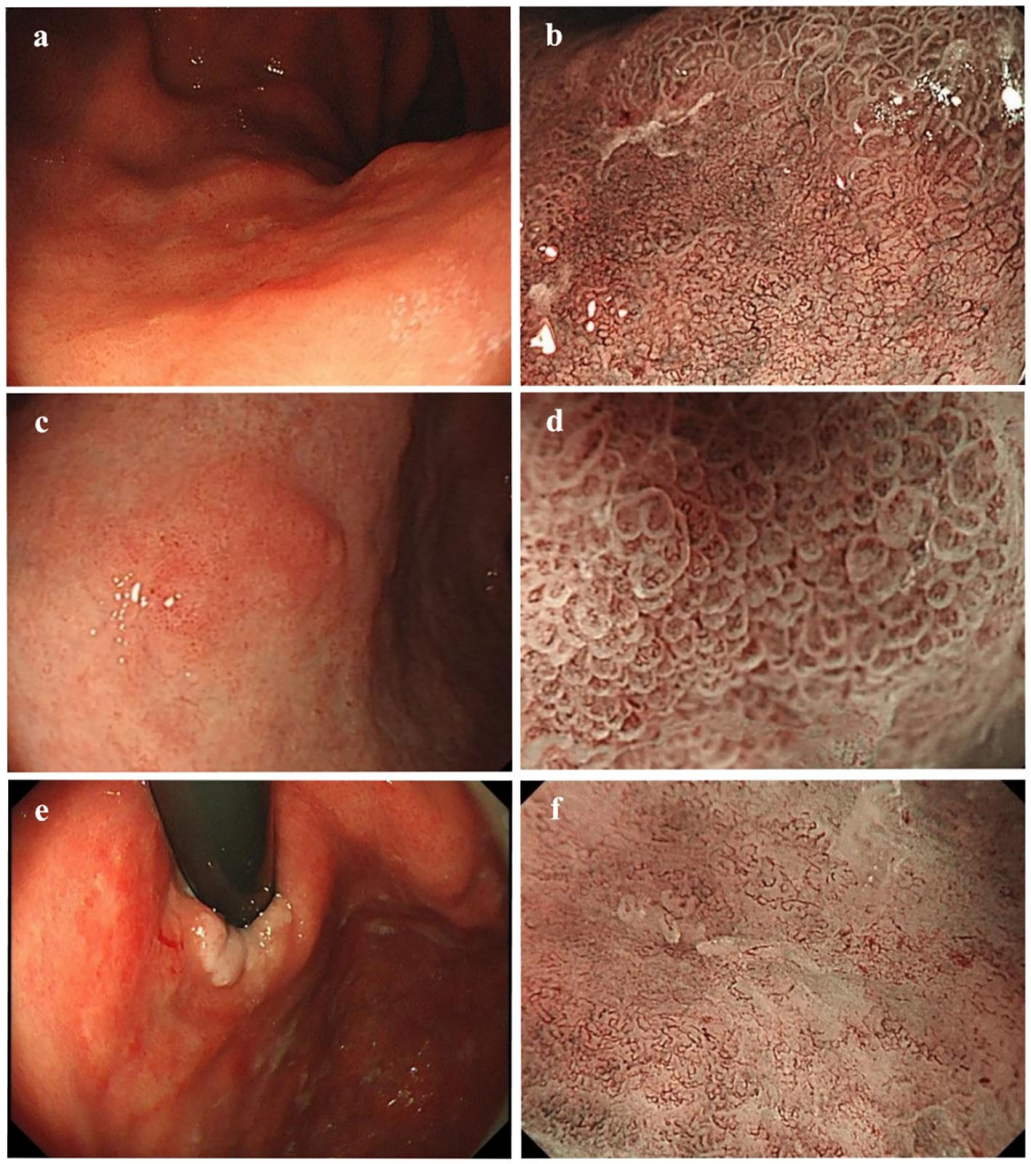
**(a, b)** White-light endoscopic and NBI magnified images of moderately differentiated tubular adenocarcinoma with a crawling growth pattern: A slightly whitish, depressed type IIc lesion is visible on the posterior wall of the gastric cardia under white-light endoscopy. The lesion has a reddish appearance, clear boundaries, an irregular shape, and measures 4.9 × 4.1 cm. The magnified NBI image shows irregular glandular ducts and visible mesh-like vessels. **(c, d)** White-light endoscopic and NBI magnified images of papillary adenocarcinoma: A slightly elevated type IIa lesion is seen on the anterior wall of the gastric angle under white-light endoscopy. It appears slightly reddish, with unclear boundaries and an irregular shape, measuring approximately 1.2 × 0.9 cm. The magnified NBI image reveals vessels within the epithelial circle (VEC), a characteristic feature of papillary adenocarcinoma. **(e, f)** White-light endoscopic and NBI magnified images of mixed-type adenocarcinoma: A superficial, flat type IIb lesion is observed on the posterior wall of the gastric cardia under white-light endoscopy. The lesion is reddish, has clear boundaries, an irregular shape, and measures approximately 5.4 × 4.4 cm. It involves the dentate line and shows minor spontaneous bleeding. The magnified NBI image reveals unclear glandular structures on the oral side of the lesion, along with visible ripple-like microvessels. .

**Figure 4:**
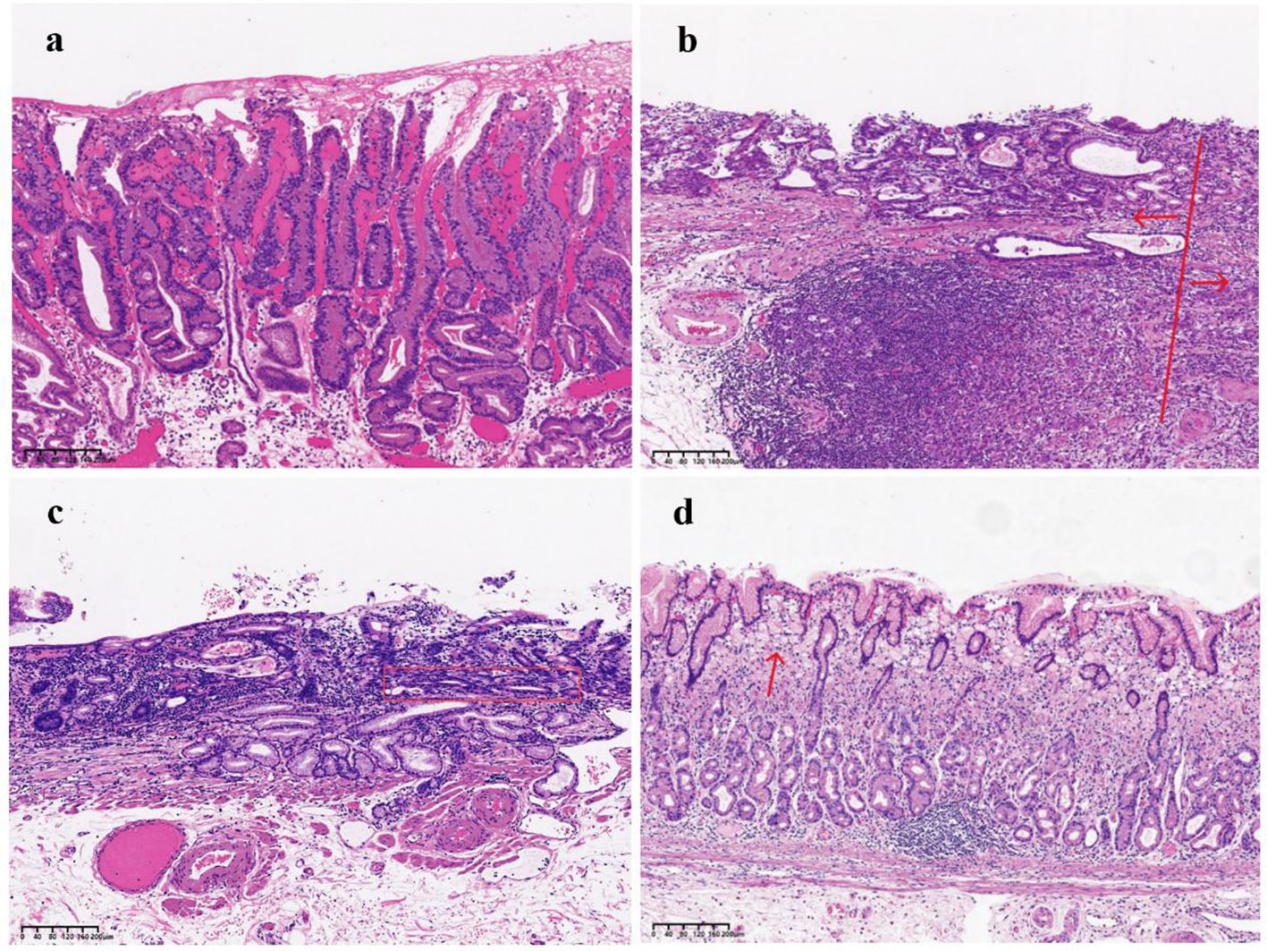
**(a)** Pap, The image illustrates numerous papillary structures, most of which are supported by a fibrovascular core. **(b)** The arrow on the left side of the image indicates a moderately differentiated tubular adenocarcinoma, while the arrow on the right side highlights poorly differentiated components. **(c)** Tub2 with a crawling growth pattern:The tumor displays moderately differentiated tubular structures, with glands forming relatively regular tubular shapes. The tubular structures, marked by the red box in the image, exhibit fusion, indicating a distinct crawling growth pattern. **(d)** SRCC: The red arrow in the image highlights signet ring cells, characterized by cytoplasm filled with mucus, which displaces the nucleus to one side.

As shown in **Table 3**, significant differences emerged in histological classification, invasion depth, surrounding mucosal intestinal metaplasia, and margin status after ESD between early GC located in different regions (all P < 0.05). Typical white-light endoscopy and NBI magnified images are presented in **Figure 3**, and magnified pathological images are displayed in **Figure 4**. Tumor diameters greater than 3 cm were more common in the early GCC group compared to the GNCC group, though the difference was not statistically significant (17.9% vs. 14.3%, P = 0.080). Tub1 was the most common histological type in both groups (55.4% vs. 50.0%). Tub2 was more frequently observed in early GCC (25.0% vs. 14.3%), while Pap (4.5% vs. 12.5%), mixed-type adenocarcinoma (15.2% vs. 16.1%), and SRCC (0% vs. 7.1%) were more prevalent in early GNCC. Additionally, severe intestinal metaplasia in the surrounding mucosa was more frequently observed in the GNCC group (49.1% vs. 78.6%, P < 0.001). Notably, early GCC was more likely to present with SM2 or deeper invasion (15.2% vs. 4.5%) and had a higher likelihood of positive margins after ESD (9.8% vs. 0.9%, P = 0.003). The incidence of lymphatic and vascular invasion was higher in early GCC compared to GNCC, although the differences were not statistically significant (lymphatic invasion: 6.3% vs. 5.4%; vascular invasion: 6.3% vs. 4.5%, both P > 0.05).

**Table 3:** Comparison of pathological features between the two groups of patients.

Pathological Parameters	Early GCC (n=112)	Early GNCC (n=112)	χ^2^	*P* value
Size, n (%)			χ^2^ = 3.075	0.080
≤3cm	92 (82.1)	96 (85.7)		
>3cm	20 (17.9)	16 (14.3)		
Histology Type, n (%)			χ^2^ = 15.870	0.003
Tub1	62 (55.4)	56 (50.0)		
Tub2	28 (25.0)	16 (14.3)		
Pap	5 (4.5)	14 (12.5)		
Mixed adenocarcinoma	17 (15.2)	18 (16.1)		
SRCC	0 (0.0)	8 (7.1)		
Depth of Invasion, n (%)			χ^2^ = 15.230	<0.001
T1a-M	79 (70.5)	102 (91.1)		
T1b-SM1	16 (14.3)	5 (4.5)		
T1b-SM2	17 (15.2)	5 (4.5)		
Intestinal Metaplasia of the Surrounding Mucosa, n (%)			χ^2^ = 21.060	<0.001
No/Mild/ Moderate	57 (50.9)	24 (21.4)		
Severe	55 (49.1)	88 (78.6)		
Margin Status after ESD, n (%)			χ^2^ = 8.805	0.003
Negative	101 (90.2)	111 (99.1)		
Positive	11 (9.8)	1 (0.9)		
Vascular Invasion, n (%)			χ^2^ = 0.352	0.553
Negative	105 (93.8)	107 (95.5)		
Positive	7 (6.3)	5 (4.5)		
Lymphatic Invasion, n (%)			χ^2^ = 0.082	0.775
Negative	105 (93.8)	106 (94.6)		
Positive	7 (6.3)	6 (5.4)		

### 3.4 Study on risk factors for SM2 or deeper infiltration in early gastric cancer

**Figure 5:**
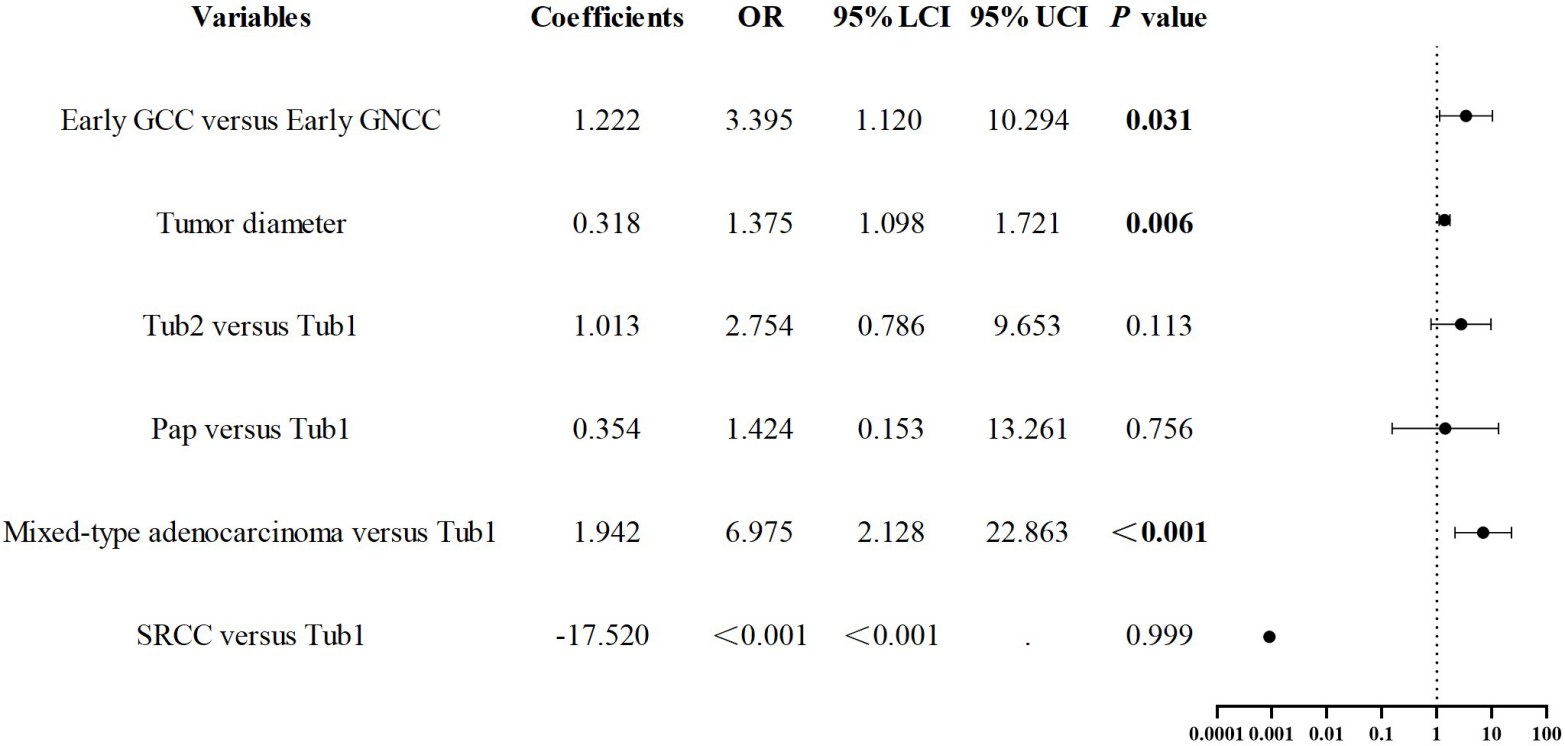
Forest plot of risk factors associated with SM2 or deeper infiltration in early GC.

After propensity score matching for early GCC and early GNCC, univariate regression analysis revealed that tumor size, location, and histological type yielded P values below 0.1. The collinearity diagnostics indicated that all VIFs were less than 2, meeting the standard criteria. These variables were subsequently incorporated into a binary logistic regression model. The analysis demonstrated that cardia location (odds ratio (OR)) = 3.395, P = 0.031), larger tumor size (OR = 1.375, P = 0.006), and mixed-type adenocarcinoma (OR = 6.975, P < 0.001) were independent predictors of early GC invasion into the SM2 layer or deeper. See **Figure 5**.

### 3.5 Study on risk factors for positive margins after ESD in early gastric cardiac cancer

**Figure 6:**
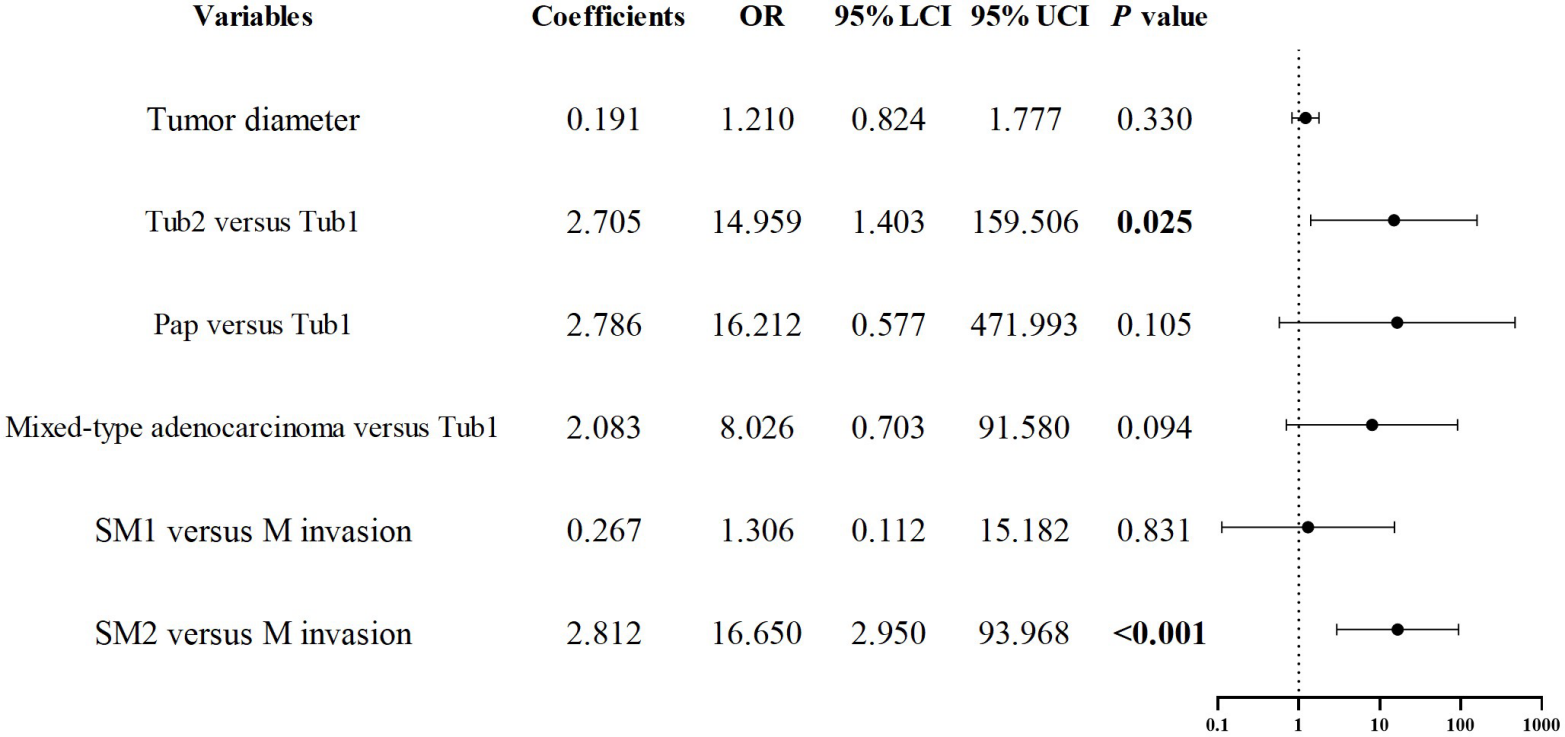
Forest plot of risk factors associated with positive margins after ESD in early esophagogastric junction cancer.

As previously noted, early GCC is more prone to deep submucosal invasion, and the incidence of positive margins after ESD is higher compared to early GNCC. To further investigate the risk factors for positive margins after ESD in early GCC, we conducted univariate regression analysis, which revealed that tumor diameter, histological type, and invasion depth were associated with statistical significance (P < 0.1). The collinearity diagnostics indicated that all VIFs were less than 2, meeting the standard criteria. These factors were included in a binary logistic regression model, and the results demonstrated that Tub2 (OR = 14.959, P = 0.025) and invasion into SM2 or deeper layers (OR = 16.650, P < 0.001) were independent predictors of positive margins after ESD. See **Figure 6** for details.

## 4 Discussion

Due to the higher incidence and unique clinical, endoscopic, and pathological features of early GCC (13–15), early GCC has garnered increasing attention from researchers. Western studies have linked the occurrence of GCC to gastroesophageal reflux disease, suggesting that it may originate from Barrett’s esophagus (16). However, Barrett’s esophagus and distal esophageal adenocarcinoma are relatively rare in China (17), with a low likelihood of progression to adenocarcinoma. This suggests that the etiology of GCC in China may differ from that in Western countries, with researchers proposing that GCC could have at least two distinct causes (18). Most previous studies comparing the pathological characteristics of gastric cancer by location have focused on advanced cases. Our study, however, analyzed the clinical, endoscopic, and pathological features of early GCC and GNCC and investigated independent risk factors for SM2 or deeper invasion and positive margins after ESD in early GCC.

Globally, the incidence of GC is approximately twice as high in men as in women. Between 2003 and 2012, the male-to-female incidence ratio for GCC ranged from 1.32 to 4.51 across different age groups, peaking between ages 60 and 64 (19, 20). In China, the male-to-female incidence ratio for GCC is higher than the global average, with two previous Chinese studies reporting ratios of 5.5 and 5.36 (12, 13), reflecting a consistent overall trend. Our findings align with previous research (3, 21, 22), indicating significant differences in GC incidence by gender and age. Specifically, early GCC is more common in elderly male patients (aged 60–69), with male incidence being 6.41 times that of females, compared to 2.49 times in the GNCC group. The higher incidence in males may be related to lifestyle factors such as smoking and alcohol consumption. Rota, Matteo (23), found that smoking increases the risk of GCC by 1.48 times compared to GNCCA similar trend was observed among men, wherein alcohol consumption was associated with a greater risk of GCC than GNCC across all categories of male drinkers (24). Several studies have also suggested that endogenous factors, such as estrogen, are associated with reduced GC incidence (25, 26), and that sex hormone-binding globulin (SHBG) in males can lower free estrogen levels, potentially promoting the development of GC (27–29). Currently, the molecular mechanisms underlying the site-specific effects of sex hormones on GC remain unclear. We aim to further elucidate the differential regulatory networks of sex hormone receptors in GCC and GNCC through an integrated approach combining prospective cohort studies with molecular biology experiments.

Previous studies indicate that tumor diameters in early gastric cardia cancer (GCC) are larger than those in early GNCC under endoscopy (14). However, two Chinese studies on early GC have drawn the opposite conclusion (6, 22). Our results reveal that tumors larger than 3 cm are more frequent in early GCC than in GNCC (19% vs. 11.8%), although the difference is not statistically significant (P = 0.08). Regarding macroscopic type (Paris classification), flat-type tumors, compared with elevated and depressed types, tend to have indistinct borders when the tumor size is small, making them more challenging to detect during early endoscopic screening. Our study revealed that flat-type tumors were more common in early GCC than in early GNCC. Although this difference was not statistically significant, it may explain why early GCC tumors are often larger when detected. Moreover, the Tub2 histological type was more prevalent in early GCC, while Pap and SRCC were more common in GNCC. We did not observe the SRCC type in early GCC, except in mixed-type tumors containing signet-ring cell components. These histological findings are consistent with previous studies (4, 22, 30). Some studies have also suggested that undifferentiated histological types are more frequent in proximal tumors (14), and the differences in study results may originate from variations in the stages of the populations included. One study found that the incidence of undifferentiated tumors in advanced GCC was significantly higher than in early GCC, suggesting that as tumors progress, GCC may evolve from differentiated to undifferentiated type (15). Since undifferentiated carcinomas are more prone to lymphatic and vascular invasion, early diagnosis and treatment of GCC are crucial. The Correa model represents a widely accepted pathway for gastric cancer development (31), with intestinal metaplasia and atrophic changes in the surrounding mucosa being key indicators. In our study, severe intestinal metaplasia was observed in the surrounding mucosa of 73.6% of GNCC cases, compared with only 51.6% of GCC cases (P < 0.001), indicating that the pathogenic mechanisms of GCC and GNCC may differ.

Submucosal deep invasion is an aggressive feature of early GC and is often associated with an increased risk of lymphatic and vascular invasion (21). SM2 or deeper invasion represents a relative contraindication for ESD and is the most common reason for non-curative resection after ESD (14). Therefore, accurate assessment of tumor invasion depth is essential. In line with findings from other studies (6, 14, 22, 30), we similarly observed that the proportion of early GCC with SM2 or deeper invasion was higher than that in GNCC (P < 0.001). Multivariate analysis identified cardia location, larger tumor diameter, and mixed-type adenocarcinoma as independent risk factors for SM2 or deeper invasion. Similarly, Koh J.S. demonstrated that larger tumors, cardia location, and lymph node invasion were risk factors for SM invasion (14). Hyeong Ho Jo et al. also demonstrated that mixed-type adenocarcinoma is associated with submucosal invasion in early GC (32). Conversely, Wang Yaqin et al.’s research suggested that tumor location is not related to invasion depth (33). Additionally, Qin Junfu et al. reported that early GC with moderately differentiated components are more likely to exhibit submucosal invasion (34). In endoscopic treatment, special attention should be given to cardia-region tumors and larger mixed-type adenocarcinomas. Accurate assessment of SM2 or deeper invasion is crucial for selecting appropriate treatment strategies (35). However, it is important to acknowledge that the conclusion of this study—that ‘GCC is more invasive’—primarily relies on evidence of deeper tumor infiltration and lacks direct prognostic data. This limitation may reduce the clinical applicability of our findings. To address this, future research will incorporate longitudinal patient follow-up and direct prognostic measures, such as survival analyses, to further validate the invasive nature of GCC and its impact on long-term patient outcomes.

Complete resection is defined as the endoscopic removal of the entire lesion with no involvement of the lateral or vertical margins on pathological examination. Given the limited follow-up duration (<10 years), this study does not provide a comprehensive analysis of patient survival rates following ESD surgery, but Koh J.S.’s study demonstrated that patients undergoing non-curative resection had lower overall survival rates (14). We found that early GCC had a higher risk of positive margins after ESD compared to GNCC, with a non-curative resection rate of 10.3% for early GCC. The non-curative resection rate was 3.5% and 5% for M and SM1 invasion, respectively, rising to 50% for SM2 or deeper invasion. This trend was also confirmed in Cao S.’s study (21). Research on the risk factors for positive margins after ESD in early GCC is limited, and in our study, only one GNCC case had a positive margin. Therefore, we performed a multivariate analysis on risk factors for positive margins after ESD in early GCC. Of 126 early GCC cases, 13 had positive margins, including 8 with positive vertical margins and 5 with positive horizontal margins. Multivariate analysis showed that Tub2 and SM2 or deeper invasion were independent risk factors for positive margins after ESD. Fu Q.Y.’s study found that tumor diameter >3 cm and mixed-type tumors were associated with positive lateral margins after surgery (35). Similarly, Koh J.S.’s study suggested that tumors ≥2 cm and undifferentiated histology were associated with non-curative resection in ESD (14). Our results indicated that tumor diameter >3 cm increased the risk of positive margins after ESD, although this increase was not statistically significant.

All five cases with positive horizontal margins had Tub2 histology, and three of these cases had a crawling growth pattern. N. Okamoto and colleagues proposed that “crawling-type” adenocarcinoma (CTAC) spreads horizontally in the mucosa, leading to the formation of poorly differentiated tissue and submucosal invasion, with a depressed gross type being common (36). The unique biological behavior of horizontal spread in CTAC could explain the positive horizontal margins after ESD. In our study, 34 early GCC cases had Tub2 histology, among which 20 had CTAC (13 were depressed type). This form of CTAC predominantly localizes to the glandular neck, with portions of its surface covered by normal epithelium. Endoscopic examination may present challenges in precisely delineating tumor boundaries, as the tumor exhibits discontinuous, skip-like growth due to low cellular adhesion. Therefore, when managing early GCC, clinicians should be cautious of Tub2 histology and consider expanding the horizontal resection range where appropriate, to reduce the risk of positive margins and the need for additional surgery.

SM2 invasion is a primary focus of our discussion and is considered a relative contraindication for ESD. Studies indicate that endoscopic ultrasound or magnifying endoscopy has an approximate 70% accuracy in assessing invasion depth (37), and in clinical practice, distinguishing between SM1 and SM2 invasion depths remains challenging. If a fully informed patient opts for ESD and is willing to accept potential subsequent surgery and associated risks, we are prepared to provide this treatment option to preserve cardia function. We also consider that if invasion depth remains unclear and the patient undergoes surgery based on our strong recommendation, postoperative pathology may reveal ESD-suitable indications, leading to irreversible tissue damage. Our follow-up results suggest the future potential to moderately expand ESD indications. Routine use of ESD as a “macro-biopsy” prior to surgery for early gastric cancer patients warrants further investigation. Additionally, subsequent treatment guided by the pathology and genetic profiling from the “macro-biopsy” may benefit patients, contingent on thorough communication and respect for patient autonomy. For elderly patients or those unfit for surgery, ESD remains an acceptable palliative option. Nonetheless, without strong evidence-based support, we acknowledge the essential role of surgery in treating early gastric cancer. For early gastric cancer with confirmed SM2 deep invasion, we continue to recommend surgery as the primary treatment.

GC remains a significant health and economic burden, and early detection and treatment are essential to reducing mortality. Previous studies indicate that early GCC has a poorer prognosis compared to GNCC (4–6). Our study found that early GCC is associated with a higher risk of deep invasion and positive margins after ESD, potentially contributing to its worse prognosis. Endoscopists should be particularly vigilant in elderly male patients for early GCC, carefully assessing histological type and invasion depth, and selecting appropriate treatment strategies. Additionally, when encountering Tub2 histology under endoscopy, horizontal margins should be carefully evaluated, and the resection area may need to be expanded to avoid positive margins.

While current research has largely defined ESD indications, international and domestic guidelines have yet to distinguish early GC by specific anatomical location. Future ESD guidelines should consider designating GCC as a distinct type of GC with specific indications. This study has several limitations. First, as a single-center retrospective study with a small sample size, its findings are constrained by the clinical practices of a single institution. Variability in endoscopists’ technical proficiency and differences in the criteria used to determine ESD indications may affect the external validity of the results. Moreover, the retrospective design may overestimate the success rate of ESD by 10–15%. Future research should incorporate multicenter prospective studies with standardized operating protocols to enhance the generalizability of these findings. Second, the current analysis primarily focuses on clinicopathological characteristics and does not incorporate molecular markers—such as HER2, EBV, or epigenetic alterations—that could elucidate the biological differences between GCC and GNCC. The absence of molecular-level data may obscure the direct impact of key oncogenic drivers on tumor phenotype. To address this, future studies should integrate high-throughput sequencing or multi-omics approaches to provide a more comprehensive mechanistic understanding.

## 5 Conclusion

Early GCC and GNCC differ significantly in clinical and pathological characteristics, with GCC generally displaying higher aggressiveness. Therefore, managing early GCC cases with suspected SM2 invasion requires a cautious, rigorous approach, including postoperative assessment for potential additional surgery and careful follow-up. Due to the unique anatomical structure of the cardia, this region has a higher risk of missed diagnoses. Enhanced endoscopic screening and differentiation of the cardia region are recommended to enable early detection and intervention while the tumor remains small, improving cardia cancer prognosis.

## Data Availability

The raw data supporting the conclusions of this article will be made available by the authors, without undue reservation.
